# Molecular and Analytical Understanding of Resveratrol Interactions for Advanced Biotechnological Applications

**DOI:** 10.3390/molecules31101747

**Published:** 2026-05-20

**Authors:** Mohamed Brahmi, Sara Moumnassi, Adem Gharsallaoui

**Affiliations:** 1 Université Lyon 1, CNRS, LAGEPP, UMR 5007, 01000 Bourg-en-Bresse, France; mohamed.brahmi@univ-lyon1.fr (M.B.); sara.moumnassi@ump.ac.ma (S.M.); 2 Laboratory of Bioresources, Biotechnology, Ethnopharmacology and Health, Faculty of Sciences, Mohammed Premier University, Oujda 60000, Morocco

**Keywords:** polyphenol-protein interactions, analytical characterization, calorimetric analysis, spectroscopic techniques, molecular modelling, computational biophysics, carrier systems, bioavailability, biotechnological applications

## Abstract

Interactions between resveratrol and biological or carrier systems play a key role in determining its bioavailability, stability, and delivery performance. These interactions involve proteins, lipids, cyclodextrins, nucleic acids, polysaccharides, and other formulation matrices, and are governed by noncovalent forces such as hydrogen bonding, hydrophobic interactions, π–π stacking, and desolvation effects. This review examines how complementary spectroscopic, calorimetric, structural, and computational techniques are used to characterize resveratrol interactions. Fluorescence, UV–visible spectroscopy, circular dichroism, FTIR, NMR, ITC, DSC, X-ray diffraction, molecular docking, and molecular dynamics simulations are discussed according to their contribution to binding analysis, conformational assessment, thermodynamic interpretation, structural organization, and complex stability. By integrating these approaches, this review provides a technique-oriented framework for understanding resveratrol binding and guiding the development of more stable resveratrol-based carrier systems and bioactive formulations.

## 1. Introduction

Resveratrol (trans-3,5,4′-trihydroxystilbene) is a polyphenolic stilbene produced as a phytoalexin in a wide range of plant species, most notably in the skins, seeds, and stems of grapes, where it accumulates in response to biotic and abiotic stress [1]. Grape pomace, the by-product of winemaking, retains a significant proportion of resveratrol and related stilbenes, making it an important and sustainable source of this compound [2].

Over the past decades, this compound has attracted substantial scientific attention due to its broad spectrum of biological activities. Numerous in vitro, in vivo, and clinical investigations have demonstrated its antioxidant [3], anti-inflammatory [4], cardioprotective [5], neuroprotective [6], and anticancer effects [7], among several other health-promoting properties [8].

Despite these promising biological attributes, the inherent physicochemical limitations of resveratrol have hindered its practical application. Its water solubility is extremely low (0.03–0.05 mg/mL), which limits oral absorption and complicates formulation in aqueous systems [9]. Following oral administration, only trace amounts of the active form are detected in the bloodstream due to extensive phase II metabolism, including glucuronidation and sulfation, leading to an oral bioavailability of less than 1% [10]. Moreover, the *trans* isomer is photosensitive and undergoes light-induced isomerization to the *cis* form. It is also susceptible to oxidative degradation under environmental conditions, which may contribute to a loss of bioactivity [11]. Oxidative degradation is particularly critical in cell culture environments, where up to 96% of resveratrol can decompose within 24 h at 37 °C, generating reactive species such as hydrogen peroxide [12]. These physicochemical and pharmacokinetic constraints severely restrict the direct therapeutic use of resveratrol and highlight the need for carrier-assisted approaches to enhance its stability, solubility, and targeted delivery [13].

To overcome these challenges, a wide range of carrier systems has been developed. The underlying concept is that encapsulation or association of resveratrol within a carrier matrix can markedly enhance its physicochemical properties and biological performance by improving stability, solubility, and bioavailability, while also enabling controlled release and site-specific delivery. Various formulation strategies have been explored, including protein-based nanoparticles, polymeric microparticles, and lipid-based carriers such as emulsions and liposomes [14].

Accordingly, the development of effective resveratrol-based formulations requires comprehensive analytical characterization to elucidate its interactions with different biological and carrier systems. These interactions, whether involving proteins, lipids, cyclodextrins, or other macromolecular matrices, play a crucial role in determining the compound’s solubility, stability, and bioavailability. Analytical tools are therefore essential for assessing the nature and strength of these interactions, monitoring structural modifications, and elucidating binding mechanisms.

Recently, several reviews have addressed different aspects of resveratrol research, yet none has adopted the technique-oriented, interaction-focused perspective developed in the present work. The biological activities of resveratrol and its potential as a dietary supplement have been extensively reviewed [15], as have the encapsulation strategies, including spray drying, liposomes, emulsions, and nanoencapsulation, designed to overcome its poor bioaccessibility and stability [14]. While these contributions provide essential context for understanding why resveratrol requires carrier-assisted delivery, they do not examine the analytical methods through which the molecular interactions underlying encapsulation and biological binding are characterized. Separately, validated chromatographic and spectrophotometric methods for trans-resveratrol quantification have been comprehensively compiled [16], covering RP-HPLC, LC-MS/MS, and related techniques across matrices including wine, serum, and nanoparticle formulations; however, this body of work addresses detection and quantification rather than the characterization of molecular interactions. At a broader level, methodological overviews of polyphenol–protein interaction techniques have provided valuable surveys of fluorescence, UV-Vis, CD, FTIR, ITC, DSC, NMR, XRD, and computational approaches [17,18], but by treating polyphenols as a unified class, these reviews did not consider how the specific physicochemical properties of resveratrol influence its interaction profile across different matrices and create analytical challenges distinct from those of flavonoids or phenolic acids. Furthermore, no review has systematically aligned the analytical, thermodynamic, structural, and computational tools currently employed to investigate these interactions, nor has it examined how each technique provides complementary mechanistic insights.

The novelty of the present review lies in addressing this gap. By integrating spectroscopic methods (UV–Vis, fluorescence, circular dichroism), calorimetric analyses (ITC), thermal and structural techniques, and in silico approaches, this work offers the first comprehensive and technique-oriented synthesis specifically focused on resveratrol. Through this analytical perspective, the review emphasizes how various methodologies reveal different aspects of resveratrol’s binding, conformational behavior, and functional implications in both biological environments and engineered carrier systems, and create analytical challenges distinct from those of other polyphenol classes, thereby providing a more mechanistic, tool-based framework for understanding and designing strategies for resveratrol-based carrier strategies.

## 2. Review Conceptualization

This review was conceptualized through a structured literature survey of major scientific databases relevant to biomedical, pharmaceutical, and food sciences, including PubMed, Scopus, and Google Scholar. The searches were conducted in September 2025, using different combinations of targeted keywords such as “*polyphenols*,” “*trans-resveratrol*,” “*interaction*,” “*protein*,” “*DNA*,” “*biopolymers*,” “*binding mechanism*,” “*spectroscopic analysis*,” “*isothermal titration calorimetry* (*ITC*),” and “in silico.” Additional relevant studies were identified through manual examination of the reference lists of selected articles. The review focused on peer-reviewed research articles reporting direct experimental or computational investigations of resveratrol binding to carrier systems or biological targets. Studies were selected when they provided mechanistic or analytical information on resveratrol interactions, including binding affinity, stoichiometry, thermodynamic parameters, conformational changes, spectroscopic shifts, structural organization, or predicted binding modes. Particular emphasis was placed on studies reporting quantitative and mechanistic descriptors, including binding affinity, stoichiometry, thermodynamic parameters, and structural or spectroscopic readouts, as these metrics are essential for elucidating molecular interactions.

Studies were not retained as core references when they focused only on resveratrol extraction, quantification, biological activity, or formulation performance without providing clear interaction or complexation data. General studies on polyphenols were also excluded when resveratrol-specific information could not be clearly distinguished.

The screening was performed in two steps. First, titles and abstracts were examined to identify studies related to resveratrol interactions with proteins, lipids, DNA, cyclodextrins, biopolymers, or carrier systems. Second, the full texts of potentially relevant articles were assessed to confirm whether they contained sufficient analytical or mechanistic information for inclusion. Foundational studies frequently cited within the field were included alongside recent publications to provide both historical context and current methodological perspectives.

The literature selection was guided by the relevance of each study to the molecular interpretation of resveratrol binding behavior and by the analytical depth of the reported data. This selection strategy ensured broad coverage while maintaining analytical depth, capturing the diversity of methodologies used to study resveratrol–biomolecule interactions while focusing on the most informative and experimentally grounded evidence for understanding the compound’s binding behavior.

## 3. Structural Basis and Molecular Behavior of Resveratrol

**Figure 1:**
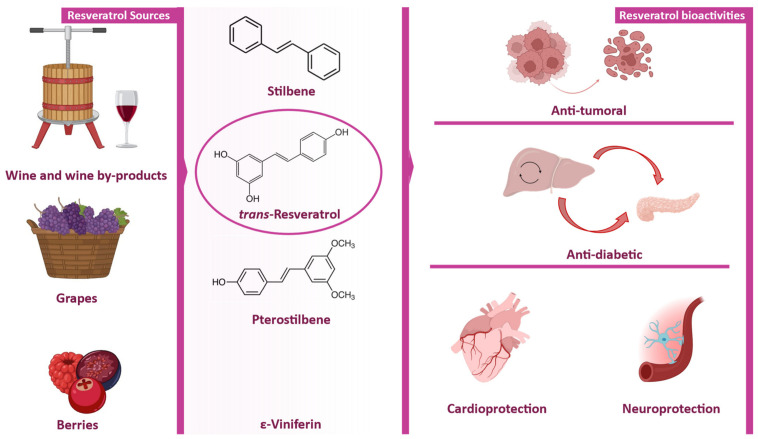
Dietary sources and biological activities of trans-resveratrol.

**Figure 2:**
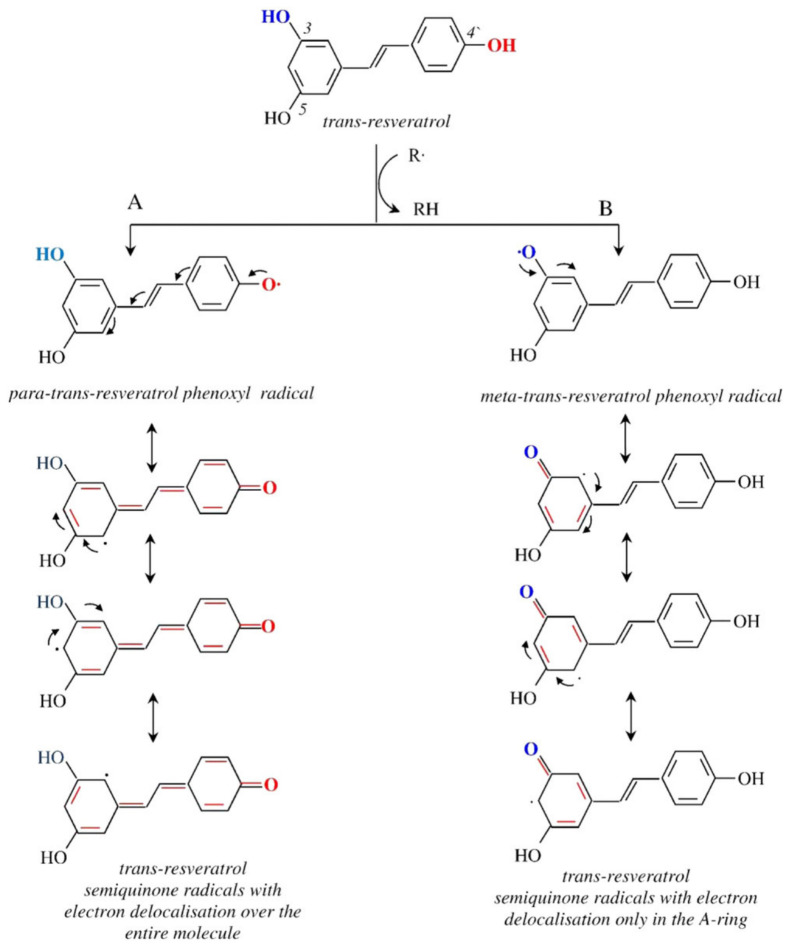
Schematic representation of free radical-scavenging activity of *trans*-resveratrol. Reproduced with permission from [35].

Resveratrol (trans-3,5,4′-trihydroxystilbene) is a stilbene derivative composed of two aromatic phenyl rings connected by an ethylenic double bond (C=C), which confers a nearly planar and rigid conformation (Figure 1). The molecule possesses three hydroxyl groups, two located on one aromatic ring and one on the other, which play a central role in determining its polarity, redox reactivity, and binding behavior [19]. Resveratrol exists in two isomeric forms (*cis* and *trans*) depending on the configuration around the central double bond, with the *trans* isomer being more energetically stable and primarily responsible for its biological activity [20]. X-ray crystallography has confirmed this nearly planar geometry and revealed a highly ordered hydrogen-bonding network among the phenolic groups [21], consistent with the molecule’s propensity for hydrogen bonding and π–π stacking. The antioxidant mechanism of trans-resveratrol, illustrated in Figure 2, exemplifies this structure–activity relationship, with the ability of its three hydroxyl groups to donate hydrogen atoms or electrons to reactive species, generating resonance-stabilized phenoxyl radicals across the conjugated aromatic system [22,23,24].

This combination of hydroxyl-mediated hydrogen bonding, aromatic planarity favoring π–π stacking, and the hydrophobic character of the stilbene backbone constitutes the molecular basis not only for antioxidant activity, but also for the broader biological profile of resveratrol, including antimicrobial, anti-inflammatory, and anticancer effects [25,26,27,28].

This versatility is evident across different biomolecular contexts. For instance, in enzyme systems, structural and computational analyses have shown that resveratrol forms stable hydrogen bonds with catalytic residues of phospholipase A_2_, with its hydroxyl groups interacting with Ser23 and adjacent amino acids to block substrate access [29], while in lipid environments, molecular dynamics simulations revealed that resveratrol localizes within the phospholipid headgroup region, where it forms hydrogen bonds with the ester carbonyls of the sn-1 and sn-2 chains, thereby enhancing membrane stability [30]. When interacting with starch, resveratrol was reported to form inclusion complexes with V-type amylose helices stabilized by hydrogen bonding with glucose hydroxyl groups and hydrophobic contacts within the helical cavity [31]. At protein interfaces, Chen et al. demonstrated that both hydrogen bonding and hydrophobic forces drive the interaction of resveratrol with whey protein isolate, promoting interfacial adsorption and reducing protein–lipid co-oxidation [32]. Finally, in nucleic acid systems, resveratrol has been shown to inhibit DNA polymerases α and δ through hydroxyl-mediated binding at catalytic sites [33], while spectroscopic and sequence-resolved studies have revealed its capacity for both intercalation into calf-thymus DNA and minor groove binding to AT-rich duplexes [34].

These structural attributes explain the ability of resveratrol to associate with chemically and structurally diverse biomolecular targets. Defining these interactions more precisely requires analytical techniques that can assess binding strength, thermodynamic parameters, conformational effects, and stoichiometry. Accordingly, the following section discusses the main analytical approaches used to study resveratrol–biological system interactions, with emphasis on their respective contributions, complementarities, and limitations.

## 4. Analytical Investigation of Resveratrol Interactions with Biological and Carrier Systems

Understanding the interactions of resveratrol with carrier materials and biological targets requires analytical approaches capable of translating molecular affinity into measurable binding parameters. While the main noncovalent forces involved in resveratrol recognition have been introduced above, their relative contribution depends strongly on the target matrix and experimental conditions. Advanced analytical techniques are therefore needed to quantify binding strength, monitor conformational changes, and distinguish between different interaction mechanisms.

Over the past decade, a comprehensive set of analytical techniques has been employed to study polyphenol–protein and polyphenol–carrier interactions. Spectroscopic methods, including ultraviolet–visible (UV–Vis) spectroscopy, fluorescence spectroscopy, circular dichroism (CD), and Fourier-transform infrared (FTIR) spectroscopy, have been widely applied to detect conformational changes and binding-induced alterations in the electronic environment [36]. In parallel, calorimetric techniques such as isothermal titration calorimetry (ITC) and differential scanning calorimetry (DSC) provide thermodynamic insights that reveal whether the binding process is primarily driven by enthalpic or entropic contributions [37]. Together, these methods provide key parameters such as binding constants, stoichiometric ratios, and heat signatures, which together help categorize interactions as weak, moderate, or strong.

Analytical investigations have also demonstrated the influence of environmental factors on the affinity and specificity of polyphenol binding to proteins, lipids, and carrier systems [17,18]. Parameters such as pH, ionic strength, temperature, and solvent composition can markedly alter interaction profiles, which is particularly relevant in formulation science where subtle variations may significantly affect solubility, stability, and bioavailability. However, it should be noted that most studies discussed in the following subsections rely on purified model systems, such as isolated proteins, defined lipid vesicles, or cyclodextrin solutions, under highly controlled laboratory conditions. Although such reductionist approaches are valuable for identifying and quantifying specific molecular interactions, they may not fully reflect the complexity of physiological or food-processing environments, where resveratrol is exposed to competitive binding, enzymatic degradation, and matrix-dependent effects.

**Figure 3:**
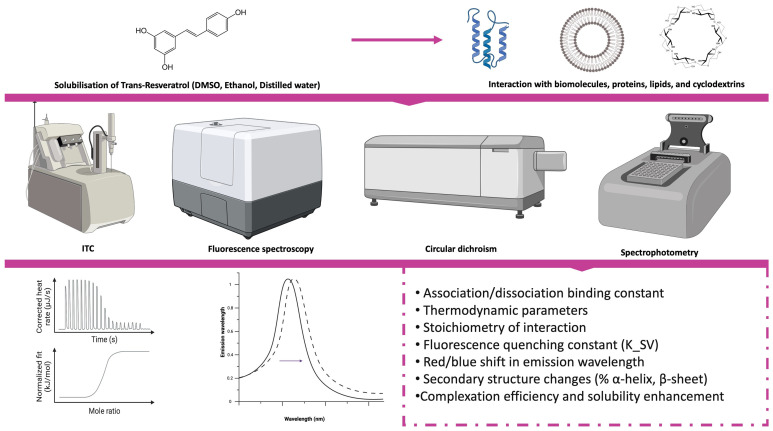
Multi-technique analytical approaches for characterizing Trans-Resveratrol interactions with biomolecules.

Building upon these methodological principles, resveratrol represents an instructive example of how analytical tools can illuminate molecular-level dynamics. The following subsections describe how these techniques have been applied to characterize the interactions of resveratrol with proteins, lipids, and carrier systems. A summary of the principal analytical strategies used to investigate resveratrol binding with various biological and physicochemical targets is provided in Table 1, while representative experimental outcomes are illustrated in Figure 3.

### 4.1. Isothermal Titration Calorimetry

Isothermal titration calorimetry (ITC) is widely recognized as an effective method for the direct, label-free quantification of molecular interactions in solution. This technique measures the heat released or absorbed during binding events, providing valuable insight into biomolecular recognition under conditions that closely approximate physiological environments. It eliminates the need for molecular labeling or immobilization [73]. Due to these advantages, ITC has been extensively applied to study interactions between small molecules, such as polyphenols, and macromolecular targets, including proteins and carrier systems.

A single ITC experiment provides a complete thermodynamic profile of an interaction, yielding the binding stoichiometry (n), enthalpy change (ΔH), entropy change (ΔS), and Gibbs free energy (ΔG). Depending on the interaction model, system stoichiometry, and thermodynamic complexity, binding affinities may be reported in different forms, as association constants (Ka), their reciprocal dissociation constants (Kd), or as stepwise and overall equilibrium constants when multiple sites or cooperative effects are present [74]. These variables are fundamental for understanding both the strength and the driving forces of molecular binding and provide mechanistic insight into the nature of the interactions involved [37].

Resveratrol poses specific challenges for ITC measurements because of its low aqueous solubility and high photosensitivity, both of which can directly influence the shape and reproducibility of thermograms. A common strategy is to dissolve resveratrol in a minimal volume of DMSO before dilution into an aqueous buffer, ensuring identical DMSO concentrations in both the syringe and the calorimetric cell [42]. This precaution minimizes mixing and dilution artifacts and is a well-established practice in ITC experiments involving hydrophobic ligands [75]. Under these conditions, binding enthalpy remains largely unaffected, although binding affinity often decreases slightly. This reduction is attributed to slower association kinetics in the more viscous cosolvent environment and a less favorable entropy term as the DMSO fraction increases, consistent with previously reported thermodynamic trends in protein–ligand systems [76].

An alternative approach avoids the use of organic cosolvents by forming cyclodextrin inclusion complexes that improve both the solubility and stability of resveratrol. In this method, resveratrol is first encapsulated within β-cyclodextrin, and subsequent ITC titrations are performed under dilute conditions [40]. Because resveratrol readily undergoes photoisomerization, producing photoproducts upon UV exposure [77], it is essential to control light exposure during sample preparation and measurement. The *trans* and *cis* isomers exhibit distinct binding affinities toward their targets, emphasizing the need for rigorous experimental control [78].

Bovine serum albumin (BSA) is frequently used as a model protein for small-molecule binding studies because of its well-characterized structure and multiple ligand-binding sites. In the ITC study by Li et al. [39], resveratrol was solubilized with β-cyclodextrin (β-CD) to enable titrations in Tris–HCl buffer (0.01 M, pH 7.4) at 298.15 K. Control experiments confirmed that β-CD did not interact with BSA under these conditions, validating the ternary experimental design. Calorimetric analysis revealed two distinct binding site classes on BSA (Table 1), one characterized by enthalpy-driven association (negative ΔH and ΔS) and the other by a significant entropic contribution consistent with hydrophobic insertion and desolvation. Notably, both BSA binding sites exhibited equilibrium constants approximately 30- to 40-fold higher than that of the β-CD/resveratrol inclusion complex measured in the same study, leading the authors to conclude that resveratrol preferentially associates with the protein rather than remaining encapsulated within the cyclodextrin cavity [39].

Further ITC investigations by Oliva et al. quantified 1:1 inclusion complexes between resveratrol and both β-CD and randomly methylated β-CD (RAMEB). While resveratrol and methyl jasmonate (MeJA) exhibited comparable affinities for β-CD, RAMEB showed marked selectivity for resveratrol, with an association constant approximately 15-fold higher than that of MeJA [40]. Competitive ITC and NMR experiments confirmed the formation of mutually exclusive binary complexes, with no evidence of ternary assemblies [40].

ITC has also been applied to investigate the binding of resveratrol to biological targets. A recent study examined its interaction with death-associated protein kinase 1 (DAPK1), a regulator of apoptosis and autophagy that plays an emerging role in the pathology of Alzheimer’s disease [42]. Experiments were conducted at 298 K in a buffer containing 40 mM HEPES (pH 7.4), 50 mM NaCl, 0.5 mM TCEP, and 0.25% DMSO. The titration of resveratrol into DAPK1 produced clear exothermic peaks fitting a one-site binding model. The titration yielded a dissociation constant in the low micromolar range (Kd ≈ 1.3 µM), with a strongly enthalpy-driven thermodynamic profile and an unfavorable entropy contribution (Table 1). Similar signatures were observed for resveratrol derivatives, suggesting that hydroxyl-mediated hydrogen bonding with the kinase hinge region represents the dominant binding mechanism, a feature of relevance to structure-based inhibitor design.

In addition to protein interactions, ITC has proven useful for optimizing encapsulation systems designed to enhance the stability and bioactivity of resveratrol. A recent study compared gelatinized wheat starch (WS) and hydrolyzed wheat starch (HWS) in their ability to form complexes with resveratrol [41]. ITC measurements were performed at 25 °C, with resveratrol solutions (200–20 μM in PBS containing 1.5% ethanol) titrated into starch dispersions (0.1–4 mg/mL in PBS with the same ethanol concentration). The resulting thermograms revealed distinct thermodynamic behaviors depending on starch type. WS–resveratrol binding was endothermic and entropy-driven (ΔS > 0, ΔH > 0), consistent with hydrophobic inclusion within amylose helices. In contrast, HWS–resveratrol interactions were exothermic and enthalpy-driven (ΔH < 0, ΔS < 0), suggesting hydrogen bonding and van der Waals stabilization. The authors concluded that hydrolysis disrupts the organized hydrophobic cavities of native starch, thereby shifting the binding mechanism from hydrophobic inclusion to hydrogen-bond-driven complexation. These differences correlated with functional improvements: both WS and HWS complexes enhanced resveratrol’s photostability and thermal stability, but HWS complexes provided greater bioavailability and antioxidant performance. ITC thus offered direct thermodynamic evidence that starch hydrolysis alters resveratrol’s binding mechanism and demonstrated how starch complexation can be exploited to improve the compound’s stability and biological efficacy.

The question that naturally arises is whether ITC alone is sufficient for drawing firm mechanistic conclusions when dealing with hydrophobic bioactive compounds such as resveratrol. A critical consideration is that the low aqueous solubility of resveratrol necessitates the use of solubilization strategies, typically involving DMSO as a cosolvent, ethanol-containing buffers, or pre-formed cyclodextrin inclusion complexes, each of which introduces additional thermodynamic contributions superimposed on the binding event of interest. These effects are not necessarily negligible. In the DAPK1 study [42], resveratrol was dissolved in 0.25% DMSO with matched cosolvent concentrations in both the syringe and the calorimetric cell. Although this precaution minimizes dilution and mixing artifacts, even low concentrations of DMSO can modify protein hydration shells and reduce the entropic penalty associated with ligand desolvation, thereby altering the apparent balance between enthalpic and entropic contributions. Wernersson et al. [76] further demonstrated that DMSO measurably affects ligand–protein association kinetics through viscosity-dependent solvent effects, suggesting that the unfavorable entropy terms reported in some resveratrol–protein systems may partly reflect cosolvent-mediated changes in the solvation environment rather than intrinsic binding thermodynamics alone.

A similar consideration applies to the wheat starch study [41], in which 1.5% ethanol was employed as a cosolvent. Although dilution heats were subtracted, ethanol can perturb the hydrogen-bonding network of the aqueous medium, potentially influencing both the magnitude and even the sign of ΔH in systems dominated by hydrogen-bonding interactions. This point is particularly relevant because the principal mechanistic conclusion of that study, namely the transition from entropy-driven hydrophobic binding in native starch to enthalpy-driven hydrogen bonding in hydrolyzed starch, relies directly on the thermodynamic signature obtained by ITC. Consequently, any cosolvent-induced perturbation of the enthalpic baseline may influence the interpretation of the binding mechanism. Additional complexity arises when cyclodextrins are used as solubilization vehicles, as in the BSA study reported in [39]. Under these conditions, the ITC thermogram reflects not only resveratrol binding to the protein but also the simultaneous dissociation of the resveratrol–cyclodextrin inclusion complex. Although control experiments indicated that β-CD itself did not interact detectably with BSA, the measured ΔH inevitably contains a contribution from the endothermic release of resveratrol from the cyclodextrin cavity. Incomplete deconvolution of this process could therefore underestimate the true exothermicity of the protein-binding event.

A second limitation, extending beyond solubilization effects, concerns data analysis itself. Systems exhibiting heterogeneous or partially overlapping binding sites are often simplified through fitting to single-site or two-site models, yielding parameters that may not fully capture the true complexity of the interaction landscape. This issue is particularly relevant for multidomain proteins such as BSA and HSA, where secondary low-affinity interactions may overlap with primary binding events and distort the apparent stoichiometry or thermodynamic profile. Collectively, these considerations indicate that although ITC provides uniquely direct access to the affinity, stoichiometry, and enthalpic–entropic balance of resveratrol binding, the resulting thermodynamic parameters must be interpreted with explicit consideration of the solubilization strategy and analytical model employed. Consequently, thermodynamic signatures reported across different studies are not always directly comparable, and complementary structural or spectroscopic techniques remain essential for robust mechanistic interpretation.

### 4.2. Spectroscopic Analysis

In addition to calorimetric methods, a wide range of spectroscopy-based analytical techniques has been extensively employed to explore the interactions of polyphenols with biological targets and carrier systems. Commonly applied techniques include UV–visible absorption spectroscopy, steady-state and time-resolved fluorescence spectroscopy, circular dichroism (CD), infrared (IR and FTIR) spectroscopy, Raman spectroscopy, and, in some cases, nuclear magnetic resonance (NMR) spectroscopy. Each of these approaches provides complementary information that can elucidate conformational changes in proteins and polysaccharides, variations in microenvironmental polarity, formation of hydrogen bonds, and site-specific binding events.

Unlike isothermal titration calorimetry (ITC), which delivers a complete thermodynamic profile of an interaction, including parameters such as ΔH, ΔS, ΔG, and Kd, spectroscopic techniques do not directly provide energetic data. Instead, they are particularly effective in detecting structural and microenvironmental alterations that accompany molecular binding. For instance, changes in chromophore environments can be monitored by UV–visible and fluorescence spectroscopy, while CD analysis reveals secondary-structure reorganization. FTIR and Raman spectroscopy detect hydrogen-bond formation and vibrational shifts indicative of specific molecular interactions. NMR spectroscopy further complements these observations by identifying changes in local chemical environments and interaction sites at the atomic level [18].

Spectroscopic methods are therefore most useful when interpreted as structural and microenvironmental probes rather than as stand-alone measurements of binding energetics. When combined with calorimetry or molecular modeling, they help connect binding affinity with conformational response and local changes around aromatic residues or vibrational groups.

#### 4.2.1. Fluorescence Spectroscopy

Fluorescence spectroscopy is highly valuable for investigating polyphenol interactions, as both ligands and many protein carriers contain intrinsic fluorophores that are highly responsive to variations in their microenvironment. Resveratrol itself can act as a fluorescent probe owing to its aromatic chromophore system. When excited at 310 nm, free resveratrol exhibits a fluorescence emission maximum at approximately 390 nm. This signal is extremely sensitive to environmental polarity and has been widely used to monitor complex formation in real time. The distinction between static and dynamic quenching is a central interpretive approach in fluorescence-based binding studies. Static quenching occurs when the fluorophore and ligand form a ground-state complex. In contrast, dynamic quenching results from transient collisions during the fluorophore’s excited-state lifetime. These mechanisms are commonly distinguished by their temperature dependence. In static quenching, the quenching constant usually decreases as the temperature rises because the complex becomes less stable. In contrast, in dynamic quenching, the quenching constant increases with temperature as molecular diffusion becomes more efficient. Another widely used diagnostic parameter is the bimolecular quenching rate constant (kq). Values above the diffusion-controlled limit of approximately 2 × 10^10^ L·mol^−1^·s^−1^ generally indicate that quenching is dominated by the formation of the ground-state complex rather than by collisional encounters [79]. Thermodynamic parameters provide an additional layer of interpretation by helping identify the main forces stabilizing the interaction. Negative ΔH and ΔS values are commonly associated with hydrogen bonding and van der Waals interactions, while positive ΔH and ΔS values are usually interpreted as evidence of hydrophobic contributions [80].

In the starch-based systems previously discussed, incorporation of wheat starch into resveratrol solutions produced marked changes in the fluorescence spectrum. The formation of the starch–resveratrol complex resulted in a strong enhancement of fluorescence intensity, indicating a decrease in polarity and a restriction in molecular rotation within the starch matrix. For hydrolyzed wheat starch (HWS), binding produced a distinct blue shift in the emission maximum from 390 to 378 nm. This effect is attributed to stronger hydrophobic interactions and hydrogen-bond formation between resveratrol and the hydrolyzed starch fragments. Temperature-dependent fluorescence experiments further revealed opposite trends for wheat starch (WS) and HWS, confirming that the two types of starch bind resveratrol through distinct molecular mechanisms [41].

In protein systems, the intrinsic fluorescence of resveratrol is less commonly exploited; instead, researchers often focus on the quenching of tryptophan fluorescence in proteins upon ligand binding. In the study by Jiang et al., resveratrol induced a concentration-dependent decrease in the intrinsic fluorescence of bovine serum albumin, with the tryptophan emission peak near 340 nm showing a progressive red shift [81]. These spectral changes indicated the formation of a resveratrol–BSA complex and a modification of the local polarity around the fluorophore. Stern–Volmer analysis provided binding constants and enabled differentiation between static and dynamic quenching processes [81].

An illustrative example of fluorescence-based analysis is the investigation of resveratrol interaction with pancreatic lipase, an enzyme involved in lipid metabolism that has been studied as a purified preparation in double-distilled water. Increasing concentrations of resveratrol progressively quenched the intrinsic tryptophan fluorescence of the enzyme, with Stern–Volmer analysis supporting a predominantly static quenching mechanism. The decrease in binding affinity with increasing temperature further indicated reduced stability of the resveratrol–lipase complex under thermal perturbation. Thermodynamic analysis supported a spontaneous and enthalpy-driven interaction, mainly stabilized by hydrogen bonding and van der Waals forces. Additional synchronous and three-dimensional fluorescence measurements showed changes around tryptophan residues, indicating that resveratrol binding modifies the local microenvironment of the enzyme and is accompanied by limited conformational rearrangement [55]. The corresponding binding constants and thermodynamic parameters are summarized in Table 1.

A comparable fluorescence approach was employed to study the interaction of resveratrol with catalase, where fluorescence titration showed progressive quenching of the emission maximum near 337 nm, with temperature-dependent Stern–Volmer behavior supporting complex formation. Synchronous fluorescence further indicated changes in the tryptophan microenvironment, illustrating the usefulness of fluorescence spectroscopy for detecting local structural perturbations during resveratrol–enzyme binding [54].

Three-dimensional fluorescence spectroscopy has also been applied to investigate resveratrol–protein interactions, offering a detailed fingerprint of the conformational state of the protein. Unlike steady-state or synchronous fluorescence, which focuses on specific emission bands, three-dimensional spectra record multiple excitation–emission pairs simultaneously, thereby providing a comprehensive representation of spectral variations. This technique enables the identification of subtle changes in peak position and intensity within regions associated with tyrosine and tryptophan residues. It also helps distinguish between local microenvironmental perturbations and larger-scale tertiary structure rearrangements. By providing this multidimensional profile, three-dimensional fluorescence serves as an additional tool for evaluating conformational alterations upon ligand binding, as illustrated in studies of resveratrol–protein complexes [53].

#### 4.2.2. Circular Dichroism (CD) Spectroscopy

Circular dichroism (CD) spectroscopy is particularly effective for studying the interactions between resveratrol and biomolecules because it provides direct information on how binding affects molecular conformation. Far-UV CD spectra (190–250 nm) are used to estimate the relative content of α-helices and β-sheets, while near-UV CD spectra (250–320 nm) probe the chiral environments of aromatic side chains and disulfide bonds. This distinction is particularly important in resveratrol-based systems. In many protein–polyphenol complexes, ligand binding does not substantially alter the overall secondary structure but induces detectable changes in the tertiary microenvironment, which can be observed in the near-UV region when combined with complementary fluorescence and UV–visible spectroscopy [82]. Conversely, when binding or co-assembly modifies protein folding, far-UV CD enables quantification of both the magnitude and direction of these changes, and established analytical protocols allow reliable evaluation of secondary-structure composition and comparison across different ligand-to-protein ratios [83].

This distinction is illustrated by two representative systems. In phosphate buffer at approximately pH 7.4, the resveratrol–β-lactoglobulin complex retained the characteristic far-UV β-sheet minimum near 216 nm, even at resveratrol concentrations up to 80 μM. However, the near-UV bands between 285 and 292 nm, associated with Trp19 and Trp61, showed a decrease in intensity. These findings indicate that the secondary structure remained largely intact, while local perturbations occurred in the tertiary environment, consistent with observations from complementary fluorescence measurements [57]. In contrast, in the case of insulin, the far-UV α-helical minima at 208 and 222 nm decreased markedly in intensity, and the near-UV signal, dominated by tyrosine and disulfide bond contributions, also weakened. This behavior suggests partial loss of helicity and rearrangement of tertiary packing. Supporting evidence from synchronous fluorescence and resonance light scattering confirmed these conformational alterations upon resveratrol binding [58].

#### 4.2.3. UV–Visible Spectroscopy

UV–Visible (UV–Vis) spectroscopy, though regarded as a conventional method, continues to serve as an effective analytical tool for observing and quantifying interactions between bioactive molecules and their targets [84]. In solution, resveratrol exhibits a characteristic UV absorption band arising from the conjugated π–π* transition of its stilbene structure [54]. In the study conducted by Camont and colleagues, UV–Vis spectroscopy was employed to characterize the isomeric behavior of resveratrol in aqueous media. The *trans*-resveratrol isomer displayed a distinct absorption maximum at 304 nm. However, after exposure to sunlight for eight hours, isomerization occurred, resulting in the formation of *cis*-resveratrol, which exhibited a shifted absorption maximum at 286 nm [11].

Beyond isomer characterization, UV–Vis spectroscopy is widely used to monitor complex formation and chemical modifications. Shifts in wavelength (bathochromic or hypsochromic) or changes in intensity (hyperchromic or hypochromic) serve as diagnostic indicators of binding events or degradation processes. For example, in the interaction between resveratrol and calf thymus DNA (ctDNA), a hypochromic effect accompanied by an isosbestic point at 286 nm was observed, supporting an intercalative mode of binding [34]. Similarly, UV–Vis spectroscopy has been applied to investigate protein–ligand interactions. In the case of ovalbumin, the addition of resveratrol induced a red shift in the absorption spectrum, suggesting increased exposure of aromatic residues to a more polar microenvironment and partial loosening of the protein tertiary structure [85]. Conversely, for the insulin–resveratrol complex, a blue shift was recorded, indicating transfer of aromatic residues to a less polar microenvironment, possibly through ligand-induced compaction or shielding by the bound resveratrol scaffold, which the authors attributed to a decrease in the number of allowed electronic transitions in insulin. At higher resveratrol-to-protein ratios, the absorption spectrum began to resemble that of free resveratrol, consistent with structural rearrangements within the insulin molecule [56].

UV–Vis spectroscopy has also been utilized to evaluate improvements in resveratrol solubility. In the presence of phosvitin, a phosphoprotein naturally occurring in egg yolk, the absorbance of resveratrol increased proportionally with phosvitin concentration, indicating enhanced aqueous solubility [51]. Comparable findings have been reported in host–guest studies involving α-, β-, γ-, and methyl-β-cyclodextrins, where the absorbance intensity of resveratrol increased in the presence of these carriers without notable wavelength shifts, consistent with inclusion complex formation [38].

From a quantitative standpoint, UV–Vis spectroscopy can also provide semi-quantitative insights into binding mechanisms. By monitoring the absorbance of free resveratrol and its variation with increasing concentrations of the interacting target, binding constants and thermodynamic parameters can be estimated through double-reciprocal plot analysis, as demonstrated for the resveratrol–ctDNA system [34].

#### 4.2.4. NMR Spectroscopy

Nuclear Magnetic Resonance (NMR) spectroscopy enables direct observation of molecular interactions in solution by monitoring changes in the chemical environment, dynamics, and spatial proximity of nuclei in both ligands and targets (e.g., proteins, lipids, cyclodextrins, etc.). Common NMR-based approaches include chemical shift perturbation (CSP), line broadening, saturation transfer difference (STD) NMR, nuclear Overhauser effect (NOE) or ROESY experiments, and diffusion-ordered spectroscopy (DOSY). NMR spectroscopy has been widely reported in the literature as a key tool for probing resveratrol interactions with biological targets and carrier systems. Its capacity to provide atomic-level information about binding sites, ligand orientation, and local microenvironments makes it highly complementary to bulk spectroscopic and calorimetric methods.

Cyclodextrin-based carriers, which are among the most extensively studied resveratrol hosts, have been investigated to elucidate their various binding mechanisms. For β-cyclodextrin–resveratrol inclusion complexes. The authors employed NMR and circular dichroism spectroscopy to assess the induced Cotton effect resulting from complex formation. Comparative analysis of ^1^H NMR and CD data yielded consistent association constants of 1988 ± 119 M^−1^ and 2242 ± 356 M^−1^, respectively. Furthermore, T-ROESY NMR experiments were crucial in verifying the theoretical orientation of resveratrol within the β-cyclodextrin cavity. The observed T-ROESY cross-peaks indicated that ring A (2 OH-ring) is oriented toward the narrow rim of β-cyclodextrin [86].

For lipid-based carriers, ^1^H NMR has been employed not only to confirm the incorporation of resveratrol but also to quantify its maximum loading capacity and spatial distribution within phospholipid assemblies. In soybean phosphatidylcholine liposomes, researchers monitored line broadening and chemical shift changes in both resveratrol and phosphocholine headgroup signals during titration. Combined 1D/2D NOESY and paramagnetic relaxation enhancement (PRE) experiments revealed that resveratrol resides near the phosphocholine headgroups rather than being deeply embedded within the acyl-chain region. This localization was confirmed through selective broadening effects induced by hexacyanochromate(III), an aqueous-phase PRE probe that cannot penetrate the lipid bilayer. The probe selectively broadened resveratrol and headgroup signals, confirming that the molecule remains near the bilayer interface. Quantitative integration of NMR signals further allowed estimation of a maximum loading capacity of approximately 5% w/w, directly linking molecular interaction data to formulation performance [87].

In a separate study, Kumar et al. investigated the interaction between resveratrol and the promoter DNA sequence d(CCAATTGG)_2_ to clarify its binding mode [88]. UV–Vis absorption spectroscopy showed pronounced hyperchromicity consistent with external, non-intercalative binding, while fluorescence titration suggested a ligand: duplex stoichiometry of approximately 2:1 and moderate affinity. Circular dichroism confirmed that the duplex maintained its canonical B-form conformation, indicating that resveratrol binding does not induce major structural distortion [88].

NMR spectroscopy provided the structural detail necessary to interpret these observations. The presence of NOE cross-peaks between resveratrol aromatic protons and base protons in AT-rich regions demonstrated minor groove binding. The absence of NOEs indicative of base-pair separation further confirmed a non-intercalative binding mode, consistent with UV–Vis data. Additionally, the preservation of intra-duplex NOE patterns supported the CD finding that the B-DNA geometry remains intact upon ligand association. Collectively, these results show that NMR not only corroborates the spectroscopic evidence for external, non-intercalative binding but also precisely localizes the interaction within the minor groove, providing essential structural context for understanding resveratrol–DNA association.

Collectively, spectroscopic techniques provide relatively cost-effective and sensitive approaches for monitoring the binding of resveratrol to biological and carrier targets. Nevertheless, several methodological limitations require careful consideration when interpreting fluorescence-derived binding parameters. A major concern is the spectral overlap between the absorption band of resveratrol (304–320 nm) and the intrinsic fluorescence region of aromatic amino acid residues, particularly tryptophan (~330–350 nm). As the concentration of resveratrol increases during titration, the ligand can progressively absorb excitation or emission light, generating inner-filter effects that produce apparent fluorescence quenching unrelated to true complex formation. This phenomenon is instrument- and geometry-dependent and may remain significant even at relatively low absorbance values. In ligand–protein binding studies, this artifact can affect Stern–Volmer plots and lead to overestimated quenching or binding constants when correction is not applied. This issue has been specifically recognized in resveratrol–protein systems. For instance, Rashtbari et al. corrected for the inner-filter effect when studying the interaction of trans-resveratrol with bovine liver catalase, since trans-resveratrol showed significant absorption at the excitation and emission wavelengths of the protein [63]. Therefore, in resveratrol binding studies, fluorescence quenching should not be interpreted solely from intensity loss unless absorbance correction, ligand background subtraction, and optical density control are clearly reported. When these precautions are absent, fluorescence data may still indicate interaction trends, but the derived binding constants should be considered semi-quantitative and, preferably, validated by complementary non-optical methods such as ITC, DSC, XRD, or NMR.

An additional limitation concerns the distinction between static and dynamic quenching mechanisms. In many studies, quenching constants exceeding the diffusion-controlled limit are interpreted as evidence of static complex formation; however, this criterion alone does not exclude the coexistence of dynamic collisional quenching. Time-resolved fluorescence measurements, when available, often reveal mixed quenching behavior that cannot be resolved by steady-state analysis alone. Consequently, binding constants obtained solely from Stern–Volmer analysis may overestimate the contribution of ground-state complex formation if dynamic effects are not properly deconvoluted.

Moreover, because resveratrol undergoes relatively rapid trans-to-cis photoisomerization and is susceptible to oxidation under UV irradiation, prolonged spectroscopic measurements may alter the effective concentration and structural state of the ligand during analysis. Even NMR, despite being among the most structurally informative spectroscopic techniques, can therefore encounter complications in peak assignment and quantification when multiple isomeric or oxidized species coexist simultaneously. Taken together, these considerations indicate that spectroscopic techniques are highly valuable as initial tools for probing resveratrol interactions, but their conclusions should ideally be validated using complementary analytical methods, particularly non-optical approaches such as ITC, to ensure robust mechanistic interpretation.

### 4.3. Differential Scanning Calorimetry (DSC)

Differential scanning calorimetry (DSC) is a valuable technique for examining the physical state, thermal behavior, and compatibility of resveratrol within different complexes and formulations by monitoring variations in heat flow during controlled heating or cooling cycles [67]. The study by Trollope et al. reported that pure crystalline *trans*-resveratrol exhibits a sharp melting endotherm in the mid−260 °C range, consistent with findings from other independent investigations [89]. This pronounced endothermic peak, corresponding to the melting of resveratrol, has been widely used as a diagnostic feature to evaluate its incorporation or interaction within composite materials and complex matrices [90,91].

DSC is particularly useful for mapping the thermal profiles of resveratrol complexes with biological macromolecules. In the case of human serum albumin (HSA), the native thermal signature displays two cooperative unfolding transitions that reflect the energetic stability of distinct domains. Upon interaction with resveratrol, this two-peak pattern shifts toward higher temperatures, accompanied by an increase in the apparent denaturation temperature. These effects provide clear thermodynamic evidence of ligand-induced stabilization of the native protein structure [66]. A comparable stabilizing effect of resveratrol has also been reported for α-lactalbumin, where the denaturation temperature increased relative to the native protein, confirming enhanced thermal resilience of the complex [67].

In another example, DSC has been employed to differentiate between simple physical mixtures and genuine host–guest inclusion complexes of resveratrol with cyclodextrins. For physical mixtures, the thermograms display additive thermal features corresponding to the individual components. In contrast, authentic inclusion complexes yield amorphous thermograms without the characteristic melting peak of resveratrol, indicating complete molecular encapsulation. These observations are consistent with complementary powder X-ray diffraction (PXRD) and NMR analyses that confirm the formation of stable resveratrol–cyclodextrin inclusion complexes [92].

Understanding the interaction between resveratrol and lipid bilayers is also essential, as membranes serve not only as structural barriers but also as dynamic components in protein function, signaling regulation, and molecular transport. Since resveratrol exerts many of its biological effects through nonspecific interactions with lipid bilayers, model membranes composed of defined phospholipids such as DMPC, DPPC, or DOPC have been widely used as simplified systems to probe these interactions. Although they do not fully reproduce the complexity of native membranes, they provide valuable thermodynamic insight into how resveratrol modulates lipid organization. DSC has therefore been extensively used to elucidate its impact on membrane thermodynamics. When incorporated into dimyristoylphosphatidylcholine (DMPC) multilamellar vesicles, resveratrol caused complete suppression of the lipid pretransition, broadening of the main transition peak, and a shift toward lower transition temperatures. These changes reflected reduced cooperativity in lipid packing and suggested that resveratrol localizes near the polar headgroup region, where it disrupts bilayer organization and introduces structural disorder [72]. Comparable effects were observed when resveratrol and its metabolite piceatannol were incorporated into phosphatidylcholine membranes, where DSC revealed decreases in both transition temperature and cooperativity, again implying preferential association with the headgroup region of the bilayer [71].

### 4.4. X-Ray Diffraction

X-ray-based analytical techniques provide direct structural information on the interactions between resveratrol and its carrier systems or biological models. Before examining complex systems, it is instructive to consider the diffraction characteristics of the pure compound. X-ray powder diffraction (XRD) analysis of crystalline *trans*-resveratrol reveals multiple sharp reflections at 2θ = 6.7°, 16.58°, 19.46°, 22.67°, 23.88°, and 28.5°, which are recognized as the characteristic peaks defining its crystalline lattice [93].

Powder X-ray diffraction (PXRD) has been widely employed to determine whether resveratrol maintains its crystalline form or becomes molecularly dispersed within a host matrix. For instance, in γ-cyclodextrin inclusion complexes, PXRD patterns differ markedly from those of simple physical mixtures and correspond to isostructural γ-cyclodextrin host–guest assemblies. Single-crystal X-ray diffraction further confirmed this arrangement, revealing a well-defined encapsulation geometry of resveratrol within the γ-cyclodextrin cavity [94]. Similar results have been reported for hydroxypropyl-β-cyclodextrin, where the PXRD patterns of inclusion complexes showed complete disappearance of the sharp crystalline peaks typical of resveratrol and the emergence of an amorphous halo. In contrast, physical mixtures exhibited a superposition of the individual crystalline patterns, indicating the absence of molecular inclusion [92].

In formulation research, the loss or attenuation of the characteristic crystalline peaks of resveratrol in XRD patterns of the final product is generally interpreted as evidence of successful encapsulation or amorphization. Such reductions in crystallinity have been consistently observed in diverse systems, including zein–propylene glycol alginate–rhamnolipid nanoparticles [95], lactose–resveratrol composites [96], and chitosan–phospholipid antioxidant formulations [97]. These transformations from crystalline to amorphous states are frequently associated with improved solubility, enhanced dispersion within the carrier matrix, and increased bioavailability, thereby underscoring the critical role of X-ray diffraction in confirming structural integration and physical stabilization of resveratrol in complex formulations.

### 4.5. Computational-Based Techniques

In silico approaches, particularly molecular docking and molecular dynamics (MD) simulations, have become essential tools for elucidating the interactions between resveratrol and biological or carrier systems. These computational strategies complement experimental data by offering atomic-level insights into binding modes, energetics, and conformational dynamics that are often inaccessible through traditional analytical methods.

Molecular docking enables the prediction of ligand orientations within receptor binding sites and the estimation of binding affinities through scoring functions. It can be conducted using blind or site-specific workflows based on structural data from the Protein Data Bank [17,98]. Widely used software such as AutoDock (version 4.0.2) Vina and DOCK 6 have been validated against experimental benchmarks and are frequently employed to model phenolic–protein interactions [99]. Docking provides detailed information on binding free energy, interaction forces, and amino acid residues involved in hydrogen bonding or hydrophobic interactions, as comprehensively reviewed by [100].

As with the experimental approaches discussed above, most computational studies of resveratrol rely on crystal structures of isolated proteins or simplified lipid bilayer models. Therefore, their predictions should be interpreted as reflecting binding behavior under idealized conditions, rather than fully representing the complexed and multicomponent environments characteristic of biological systems. Moreover, computational methods should be interpreted according to the extent to which they are supported by experimental evidence. When docking or MD simulations are consistent with ITC, fluorescence, CD, or NMR data, they can strengthen mechanistic interpretation by identifying likely binding residues, ligand orientations, or conformational changes. However, when used without experimental constraints, their outputs remain hypothetical and should not be treated as direct evidence of binding affinity or mechanism. This distinction is particularly important for resveratrol systems, where flexibility, solvation, and weak-to-moderate binding can cause computational models to overestimate binding strength or favor overly rigid binding poses. A summary of representative computational analyses of resveratrol interactions with different proteins and carrier systems is presented in Table 2.

**Figure 4:**
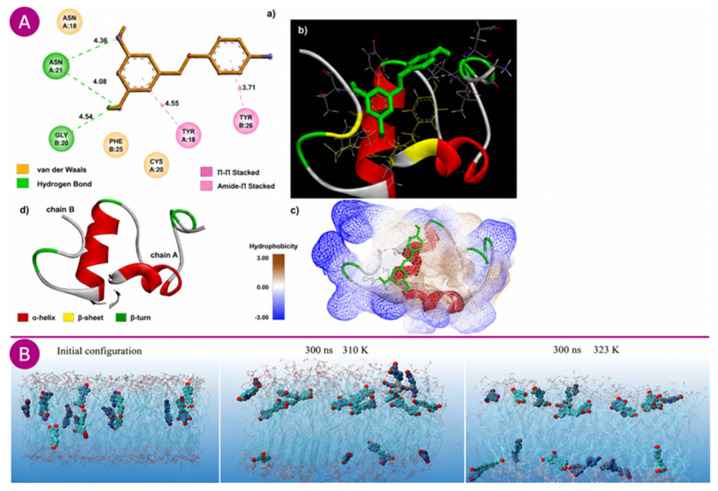
Computational insights into resveratrol interactions with biomolecular targets. (**A**) Visualization of resveratrol bound to insulin, showing the positioning of aromatic and polar residues involved in the complex (**a**): binding residue insulin-resveratrol, (**b**): binding residue with insulin secondary structure, (**c**): binding site insulin-resveratrol with hydrophobicity surface region, and (**d**): secondary structure of insulin). (**B**) Spatial distribution of resveratrol molecules within a dipalmitoylphosphatidylcholine (DPPC) lipid bilayer obtained from molecular dynamics simulations, highlighting interfacial localization near the headgroup region. Reproduced with permission from [30,56].

Docking analyses involving casein proteins revealed distinct binding affinities and residue interactions for α- and β-caseins. Bourassa et al. combined fluorescence spectroscopy, FTIR, CD, and molecular docking to characterize resveratrol binding to both caseins, reporting experimental binding constants of 1.9 × 10^4^ M^−1^ for α-casein and 2.3 × 10^4^ M^−1^ for β-casein [101]. The docking analysis, performed within this experimentally constrained context, positioned the ligand near Leu-142, Phe-150, Pro-147, and Tyr-144 in α-casein, while engaging Asn-7, Leu-3, Leu-88, Phe-87, Phe-119, Pro-115, and Val-116 in β-casein, underscoring subtle structural differences in binding sites. However, the docking-derived binding free energies (−11.56 and −12.36 kcal/mol for α- and β-casein, respectively) substantially overestimate the experimental affinities, which correspond to ΔG values of approximately −5.8 and −5.9 kcal/mol. This discrepancy illustrates that, although docking can provide useful insight into possible binding sites and interaction residues, quantitative energies obtained from docking scoring functions should be interpreted with caution [101]. Similar docking analyses on whey proteins provided complementary mechanistic insights. In β-lactoglobulin (β-LG) (Figure 4A), resveratrol was located within a hydrophobic cavity, interacting with Glu-44, Thr-18, Tyr-20, and Tyr-42, forming a π–π stacking interaction with Tyr-20 and six hydrogen bonds. In α-lactalbumin (α-LA), resveratrol occupied a hydrophobic pocket composed of Gln-54, Thr-33, Trp-104, Leu-105, and Tyr-103, establishing a π–π stacking interaction at Tyr-103 and three hydrogen bonds (Figure 4B). Comparative analysis indicated that α-LA formed a greater proportion of hydrophobic interactions, illustrating the adaptability of resveratrol’s binding modes across different whey proteins [32].

These binding characteristics provide a clear structural explanation for the DSC observations discussed previously. Binding to α-LA or β-LG predominantly involves flexible hydrophobic regions rather than deep, pre-organized binding pockets; ligand insertion into such dynamic environments perturbs local packing and alters domain mobility, resulting in modest stabilization of early unfolding transitions but destabilization of later α-helical structures, particularly in α-LA. In contrast, the stabilization observed for HSA in DSC arises from resveratrol binding to Sudlow’s site I, which is a well-defined internal cavity that restricts conformational flexibility. The docking results therefore support the interpretation that the rigidity and structural definition of the binding site largely deter-mine whether resveratrol induces protein stabilization (as in HSA) or domain-selective destabilization (as in β-LG and α-LA).

Docking has also been instrumental in elucidating host–guest inclusion complexes of resveratrol with cyclodextrins. Simulations of β-cyclodextrin and hydroxypropyl-β-cyclodextrin revealed that resveratrol predominantly interacts through hydrophobic contacts within the cavity. Modeling of axial orientations indicated that β-cyclodextrin accommodates only part of the aromatic rings, leaving hydroxyl groups exposed, whereas hydroxypropyl-β-cyclodextrin allows deeper insertion of one ring with the second oriented toward the cavity entrance [110]. Later refinements using docking and MD simulations positioned resveratrol within the cyclodextrin cavity with a calculated binding energy of −23.25 kcal·mol^−1^. Three hydrogen bonds were identified between the phenolic hydroxyl groups and the rim hydroxyls of the host, with the ligand extending slightly beyond the cavity depth, indicating partial embedding stabilized through dynamic interactions [104].

While molecular docking provides valuable structural hypotheses about resveratrol binding, its quantitative predictions warrant critical scrutiny when compared with experimentally derived binding parameters. A recurrent limitation is that docking scoring functions, which approximate binding free energies through empirical or force-field-based terms, can overestimate or misrepresent true affinities because they do not fully account for protein flexibility, explicit solvation, or entropic penalties associated with ligand immobilization. This discrepancy is evident in several systems reviewed here. For β-cyclodextrin, docking predicted a binding energy of −23.25 kcal/mol [104], whereas ITC measurements yielded a much weaker association constant (Ka = 2415 M^−1^) [40], indicating a substantial overestimation of binding strength. Similarly, MM/PBSA calculations for the resveratrol–β-lactoglobulin complex predicted a binding free energy of −27.57 kcal/mol [102], while fluorescence studies reported binding constants in the range of 10^4^–10^5^ M^−1^ [46,49], again suggesting weaker experimental affinities than those predicted computationally. Although MM/PBSA incorporates solvation and energetic corrections beyond standard docking, it remains an endpoint method and may still overestimate absolute binding affinities, particularly for flexible ligands in shallow or dynamic binding sites. An additional complication arises from inconsistencies in how binding energies are reported across the literature. For instance, the docking energies for whey proteins reported by Chen et al. [32] lack an explicit sign convention, making direct comparison with other studies difficult.

These discrepancies do not diminish the structural value of computational approaches; the identification of binding residues, interaction types, and relative orientation preferences remains highly informative and often agrees with experimental observations. However, they caution against interpreting docking-derived binding energies as quantitatively reliable estimates of affinity. For this reason, docking is increasingly complemented by MD simulations, which allow the system to evolve over time and provide dynamic metrics, such as RMSD, RMSF, Rg, and hydrogen-bond persistence, that more closely reflect the stability and behavior of resveratrol complexes in solution. Even so, MD simulations also remain dependent on force-field approximations, particularly in the treatment of polyphenol–water interactions and partial charge assignments on hydroxylated aromatic systems, which remain active areas of methodological development [100].

Molecular dynamics simulations have been extensively employed to assess the stability and structural behavior of resveratrol-based complexes over time. For the resveratrol–β-lactoglobulin system, MD simulations confirmed the stability of the resveratrol–β-LG complex over the simulation trajectory, with MM/PBSA analysis indicating a favorable binding free energy. The protein maintained its overall tertiary fold, with structural metrics (RMSD, Rg, SASA) indicating a slightly more compact conformation and reduced solvent exposure upon ligand binding. Multiple hydrogen bonds between resveratrol’s hydroxyl groups and cavity-lining residues were maintained throughout the trajectory, consistent with the integration of the ligand into the protein’s hydrophobic interior [102].

Expanding from proteins to lipid systems, MD simulations have also explored resveratrol’s behavior within lipid bilayers (Figure 4B). In dipalmitoylphosphatidylcholine (DPPC) membranes, resveratrol was positioned just beneath the headgroup region, maintaining an interfacial orientation rather than deeply penetrating the hydrophobic core. On average, the molecule formed 1.6 hydrogen bonds with phosphate or ester oxygen atoms of the lipid headgroups. Structural analyses indicated a slight increase in the area per lipid, a decrease in bilayer thickness, and reduced acyl-chain order-changes that correlate with a decrease in the main phase transition temperature. These effects suggest that resveratrol locally relaxes lipid packing while preserving the overall structural integrity of the bilayer, consistent with experimental calorimetric and spectroscopic data [30].

Collectively, computational methods transform structural hypotheses into testable mechanistic models by identifying plausible binding sites, characterizing intermolecular forces, and predicting the stability of complexes under physiological conditions. Results from molecular docking and MD simulations have shown excellent agreement with experimental data, reinforcing the predictive and interpretative power of computational approaches in elucidating resveratrol–biomolecule interactions [100,101].

## 5. Multi-Technique Convergence and Mechanistic Interpretation

The analytical techniques discussed throughout this review each capture different aspects of resveratrol’s binding behavior, but their real interpretive value emerges when results from independent methods are examined together within the same molecular system. Comparing findings across techniques not only strengthens mechanistic conclusions when observations converge, but also exposes condition-dependent subtleties that no single method can reveal on its own.

Among the systems examined, human serum albumin offers one of the clearest examples of why this integrative approach matters, largely because independent studies do not arrive at identical conclusions. Fluorescence quenching experiments consistently describe a static binding mechanism centered around the Trp-214 microenvironment, with reported binding constants ranging from approximately 10^4^ to 10^7^ M^−1^ depending on the experimental conditions [48,59]. Molecular docking studies support this interpretation by positioning resveratrol within Sudlow’s site I in subdomain IIA, where the ligand is stabilized through hydrogen bonding and van der Waals interactions involving residues such as Trp-214 [62]. However, when circular dichroism data are compared across studies, the interaction appears considerably more complex. Qureshi and Javed [62] observed an increase in α-helical content from 61.4% to 68.2% at higher resveratrol-to-protein ratios (1:2 and 1:4), together with an increase in melting temperature from 63.8 °C to 66.3 °C, suggesting ligand-induced stabilization of the protein structure. By contrast, Lu et al. [76] reported a marked decrease in α-helical content from 49.7% to 40.3% at a 1:2 molar ratio and proposed a binding location at the interface between subdomains IB and IIA rather than within the canonical Sudlow’s site I cavity. These contrasting CD observations, obtained for the same protein under different concentrations, buffer systems, and docking conditions, suggest that resveratrol–HSA interactions cannot be reduced to a single fixed binding mode. Instead, the interaction appears highly sensitive to ligand concentration, protein conformation, and solvent environment, all of which influence both binding geometry and the resulting structural response. This level of complexity would remain largely invisible if the system were examined using only a single analytical technique. The DSC results reported by Stirpe et al. [66], which showed upward shifts in both denaturation transitions (Tm_1_ from 56.9 to 58.2 °C and Tm_2_ from 64.6 to 67.1 °C) together with increased unfolding enthalpy, are thermodynamically consistent with the stabilization scenario proposed by [62], yet they appear difficult to reconcile with the destabilizing CD profile described by [76]. Collectively, these studies highlight the importance of carefully reporting and comparing experimental conditions when integrating data from multiple techniques, a practice that remains inconsistent across the resveratrol–protein interaction literature.

In contrast, bovine serum albumin presents a far more coherent multi-technique picture. ITC measurements identified two classes of binding sites with equilibrium constants of 2.69 × 10^4^ and 3.94 × 10^4^ M^−1^, indicating contributions from both enthalpy-driven and entropy-driven processes [39]. Fluorescence spectroscopy independently confirmed the formation of a stable ground-state complex at the tryptophan site through static quenching [81], while circular dichroism studies consistently showed that BSA largely preserves its α-helical secondary structure after resveratrol binding [39,49,60]. The agreement among thermodynamic analysis by ITC, site-specific binding information from fluorescence, and global structural monitoring by CD supports a model in which resveratrol associates primarily with surface-accessible hydrophobic regions of BSA without penetrating deeply enough to disrupt the overall fold of the protein. This interpretation is also consistent with the broader understanding that BSA, owing to its multidomain architecture, can accommodate small polyphenolic ligands at relatively superficial binding sites while maintaining conformational stability [80].

Cyclodextrin-based carrier systems provide another strong example of methodological convergence, particularly between solution-phase and solid-state techniques. ITC established a 1:1 stoichiometry for complex formation and differentiated the thermodynamic signatures of β-cyclodextrin and γ-cyclodextrin complexes, with β-cyclodextrin binding appearing predominantly enthalpy-driven and γ-cyclodextrin exhibiting mixed enthalpic and entropic contributions [38]. NMR spectroscopy, especially T-ROESY experiments, added structural information inaccessible to calorimetry by showing that the resorcinol ring of resveratrol preferentially orients toward the narrow rim of β-cyclodextrin [86]. Powder X-ray diffraction independently confirmed true molecular inclusion through the disappearance of the characteristic crystalline reflections of free resveratrol in authentic inclusion complexes, whereas these reflections remained visible in simple physical mixtures [92,94]. Molecular docking and molecular dynamics simulations further supported this structural arrangement by predicting partial insertion of the stilbene scaffold into the cyclodextrin cavity, stabilized by hydrogen bonding between hydroxyl groups and the cyclodextrin rim [104]. What makes this system particularly compelling is that calorimetry, NMR, diffraction, and computational modeling each probe fundamentally different physical properties, namely binding energetics, atomic proximity, crystalline order, and conformational dynamics, yet all converge toward the same structural model of inclusion complex formation.

Lipid bilayer systems offer a similarly coherent picture, with DSC, NMR, and molecular dynamics simulations all pointing toward an interfacial localization of resveratrol within membranes. Calorimetric studies on DMPC and DPPC bilayers consistently showed suppression of the lipid pretransition together with broadening and lowering of the main phase transition temperature, indicating reduced cooperativity after resveratrol incorporation [71,72]. These thermal effects are characteristic of molecules that perturb lipid packing near the headgroup region without deeply penetrating the hydrophobic core. NMR paramagnetic relaxation enhancement experiments confirmed this interpretation by demonstrating selective broadening of resveratrol signals in the presence of aqueous-phase probes, indicating that the molecule remains close to the phospholipid headgroups rather than within the acyl chain interior [87]. Molecular dynamics simulations of DPPC membranes reproduced these observations remarkably well: resveratrol maintained an interfacial orientation, formed an average of 1.6 hydrogen bonds with phosphate and ester oxygen atoms, and induced a slight increase in area per lipid together with reduced bilayer thickness [30]. The close agreement between thermal measurements, NMR structural data, and computational trajectories makes membrane systems one of the strongest examples of cross-technique validation in the resveratrol interaction literature.

When considered as a whole, these comparisons reveal several key patterns. First, when multiple independent techniques are applied to the same system under comparable conditions, the resulting conclusions generally converge toward a coherent mechanistic model. This has been observed for BSA, cyclodextrin inclusion complexes and lipid bilayers. Second, when discrepancies emerge, as illustrated by the HSA-resveratrol literature, they often reflect the sensitivity of resveratrol interactions to experimental variables rather than methodological inconsistency. Differences in ligand concentration, solvent composition, protein conformation, optical correction and the specific structural level probed by each technique can lead to divergent interpretations. Third, mechanistic interpretation must remain system-specific. Protein binding, cyclodextrin inclusion, starch complexation, lipid bilayer partitioning and DNA association do not follow the same physicochemical assumptions. In cyclodextrin, starch, lipid or cosolvent-containing systems, solvent reorganisation, cavity desolvation, hydrophobic partitioning, hydrogen bonding and matrix flexibility may contribute differently to the observed thermodynamic and spectroscopic responses. Therefore, future studies that systematically vary experimental conditions while integrating multiple analytical techniques within the same experimental framework will be essential for developing a more predictive understanding of resveratrol interactions across diverse molecular environments.

## 6. Conclusions, Limitations and Future Perspectives

This review highlights that the interaction behavior of resveratrol is governed by a variable balance of noncovalent forces, mainly hydrogen bonding, hydrophobic interactions, π–π stacking, and desolvation effects. The relative contribution of these forces depends strongly on the target system, the local microenvironment, and the experimental conditions. Therefore, resveratrol binding cannot be described by a single universal mechanism. Rather, it should be considered a context-dependent molecular process associated with solubility, molecular flexibility, isomeric state, target structure, and the presence of competing interactions.

The reviewed studies also show that the main value of analytical characterization is not only to confirm that resveratrol binds to a given protein, lipid, cyclodextrin, polysaccharide, or nucleic acid, but to clarify how this binding modifies its behavior. This point is consistent with recent reviews on resveratrol delivery systems, where the major challenge is no longer only to encapsulate resveratrol, but to demonstrate improved stability, bioaccessibility, bioavailability, and release behavior in relevant carrier matrices. In this sense, binding constants, quenching parameters, thermodynamic signatures, diffraction patterns, or docking scores should not be interpreted as isolated endpoints. Their real significance depends on whether they can explain functional outcomes such as improved solubility, protection against degradation, controlled release, cellular uptake, or modulation of biological activity. The available evidence confirms the importance of combining complementary analytical, thermodynamic, spectroscopic, structural, and computational approaches. Calorimetric techniques, particularly ITC, provide direct information on binding affinity, stoichiometry, and enthalpic or entropic contributions, but their interpretation may be affected by cosolvents, ethanol-containing buffers, or cyclodextrin-assisted solubilization required to overcome the poor aqueous solubility of resveratrol. Fluorescence spectroscopy remains one of the most widely used techniques for monitoring resveratrol–protein interactions, but fluorescence-derived binding parameters may be associated with spectral overlap, inner-filter effects, ligand autofluorescence, and the coexistence of static and dynamic quenching mechanisms. CD, FTIR, NMR, DSC, and XRD provide valuable information on conformational changes, vibrational signatures, molecular organization, thermal behavior, and crystalline-to-amorphous transitions, but these techniques also face limitations related to sample complexity, weak or transient interactions, isomerization, and the coexistence of several molecular species in solution. Computational methods are highly useful for identifying possible binding geometries, interaction residues, and conformational trends, but docking scores or simulated binding energies should be considered supportive rather than conclusive unless validated by experimental data.

Another important limitation is the predominance of simplified model systems. Most available studies use isolated proteins, model lipid bilayers, cyclodextrin solutions, starch dispersions, or short DNA sequences under controlled laboratory conditions. These systems are necessary for identifying specific mechanisms, but they do not fully reproduce the complexity of biological fluids, food matrices, gastrointestinal environments, or multicomponent carrier systems. This limitation has also been emphasized in reviews on polyphenol–lipid and oral delivery systems, where simplified membrane or digestion models are considered useful but insufficient to predict behavior in epithelial, food, or physiological environments. Under real conditions, resveratrol may undergo competitive binding, enzymatic transformation, oxidation, trans-to-cis isomerization, or redistribution between aqueous, lipid, and protein-rich phases. As a result, interaction parameters obtained in simplified systems cannot always be directly translated to food, nutraceutical, pharmaceutical, or biomedical contexts.

Future research should therefore move from the simple demonstration of complex formation toward the evaluation of functional relevance. For carrier systems, the central question is not only whether resveratrol interacts with proteins, lipids, cyclodextrins, or polysaccharides, but whether this interaction improves loading capacity, storage stability, photochemical protection, release behavior, bioaccessibility, and biological efficacy. A strong interaction may improve protection but may also restrict release, whereas a weak interaction may favor release but provide insufficient stabilization. Future studies should therefore connect molecular interaction data with functional assays, including stability during storage and processing, resistance to light and oxidation, simulated gastrointestinal digestion, release kinetics, cellular uptake, and biological activity after digestion or delivery. Greater attention should also be given to application-relevant matrices. In food and nutraceutical systems, resveratrol interactions should be evaluated in the presence of proteins, lipids, carbohydrates, salts, digestive enzymes, bile components, and processing conditions that may modify its stability and partitioning. In biomedical contexts, interaction studies should be more closely linked to serum binding, metabolism, pharmacokinetics, cellular response, and toxicity. This direction is important because several reviews on resveratrol delivery and polyphenol–protein interactions emphasize that molecular interactions may alter not only solubility and stability, but also digestibility, sensory properties, cellular uptake, and biological activity. Such studies would help distinguish interactions that are only detectable under idealized analytical conditions from those that genuinely contribute to improved delivery, bioavailability, or biological function.

Methodological standardization remains necessary, but it should support this broader translational objective. Future studies should report solvent composition, light exposure, pH, ionic strength, temperature, optical-density limits, inner-filter correction procedures, ligand stability controls, and fitting models more systematically. These parameters strongly influence the apparent binding mechanism of resveratrol and currently limit comparison between independent studies. Applying multiple techniques to the same system under matched experimental conditions would also improve mechanistic reliability. For example, fluorescence may reveal local microenvironmental changes, ITC may define the thermodynamic signature, CD or FTIR may detect conformational effects, DSC and XRD may confirm thermal or physical stabilization, and molecular modeling may help rationalize the interaction sites. When these approaches are integrated within the same experimental design, the resulting interpretation becomes more robust than when isolated values are compared across unrelated studies.

Overall, the current literature demonstrates that resveratrol interaction research has progressed from simple binding detection toward more detailed molecular interpretation. However, the next step should be to connect this molecular understanding with practical outcomes. A more complete evaluation of resveratrol interactions requires not only analytical precision, but also functional validation in realistic biological, food, and formulation environments. Such an integrated approach will be essential for designing more effective resveratrol-based carrier systems and for clarifying how molecular interactions contribute to its stability, delivery, bioavailability, and biological activity.

## Figures and Tables

**Table 1:** Summary of resveratrol–target interactions characterized by different analytical approaches. The table compiles representative studies employing spectroscopic, calorimetric, and structural techniques to investigate resveratrol binding with proteins, lipids, and carrier matrices. Reported parameters include binding constants, thermodynamic descriptors (ΔH, ΔS, ΔG), and conformational effects, providing a comparative overview of methodologies and their interpretative value in elucidating resveratrol’s interaction mechanisms.

Analytical Techniques	Carrier/Target	Key Results	Ref
**ITC**	Cyclodextrins: α-CD, β-CD, γ-CD, methylated-β-CD (Mβ-CD)	All complexes showed a 1:1 stoichiometric ratio. Binding is mainly enthalpy-driven for α-CD, β-CD, and Mβ-CD, whereas γ-CD exhibits contributions from both enthalpy and entropy. Among the cyclodextrins, γ-CD displays the highest affinity expressed as equilibrium constants of 4.74 × 10^4^ M^−1^, accompanied by a positive entropic contribution (TΔS = 25.2 kJ/mol)	[38]
	BSA vs. β-Cyclodextrin (competitive binding)	BSA binding was observed at two separate sites with affinities that exceeded those of β-CD. The interactions of BSA were more pronounced with two equilibrium constants describing two classes of binding (K°_1_ = 2.69 × 10^4^ M^−1^; K°_2_ = 3.94 × 10^4^ M^−1^) in comparison to the 1:1 β-CD complex (K° = 940 M^−1^), suggesting that the binding of BSA is considerably more robust than the inclusion of β-CD	[39]
	β-CD or RAMEB with RSV + MeJA (competitive binding)	ITC and NMR revealed two competing binary complexes (RSV/β-CD and MeJA/β-CD), with no ternary complex detected. For β-CD, both complexes formed with similar affinity and coexisted in comparable amounts. For RAMEB, RSV showed significantly stronger binding than MeJA, resulting in dominant RSV complex formation	[40]
	Wheat starch (WS) and hydrolyzed wheat starch (HWS)	The binding of WS–resveratrol is characterized as endothermic and driven by entropy, resulting from its encapsulation within a hydrophobic cavity. Conversely, the binding of HWS–resveratrol is exothermic and driven by enthalpy, which involves robust hydrogen-bond networks measuring 2.0–2.2 Å.	[41]
	DAPK1 kinase domain	Resveratrol binds to DAPK1 with dissociation constant (Kd ~1.3 µM), enthalpy-driven interaction mainly via hydrogen bonds with hinge region, competitively inhibits ATP binding and ATPase activity.	[42]
	HPV33 E2 DNA-binding domain	The binding process is exothermic and primarily driven by enthalpy (ΔH = −59.8 kcal/mol; ΔG = −6.28 kcal/mol; ΔS = −195 cal/mol.K; Kd = 14.7 µM). Resveratrol interacts close to the α2-helix/dimer interface, which aids in stabilizing the protein	[43]
	Superoxide dismutase	The binding process is spontaneous and exothermic (binding constant Kb = 7.3 × 10^4^ M^−1^; ΔH = −47.8 kJ/mol; ΔG = −39.2 kJ/mol; ΔS = 0.29 kJ/mol.K). Resveratrol establishes hydrogen bonds with GLU100, PRO28, and LYS23, while also participating in π–π stacking interactions with TRP32, which leads to the stabilization of the SOD1 dimer	[44]
	1-palmitoyl-2-oleoyl-sn-glycero-3-phosphocholine, (POPC), and 1-palmitoyl-2-docosahexaenoyl-sn-glycero-3-phosphocholine, (PDPC)	Resveratrol exhibits a stronger binding affinity to PDPC with equilibrium constant (KR = 1.01 × 10^5^ M^−1^) compared to POPC (KR = 1.03 × 10^4^ M^−1^), with both binding interactions being influenced by entropy (TΔS = 23.4 and 17.9 kJ/mol, respectively). The binding process is mainly governed by hydrophobic interactions and desolvation, indicating a greater preference for polyunsaturated lipids over monounsaturated ones.	[45]
**Fluorescence spectroscopy**	Whey proteins: lactoferrin, holo-lactoferrin, apo-lactoferrin, whey protein isolate (WPI), β-Lactoglobulin-rich fraction, α-lactalbumin-rich fraction	Resveratrol formed 1:1 complexes with every protein tested, exhibiting binding constants between approximately 1.7 × 10^4^ and 1.2 × 10^5^ M^−1^. The strongest binding was noted with WPI and β-lactoglobulin, while the weakest was observed with apo-lactoferrin. There were no significant alterations in the secondary structure, indicating that the binding occurs primarily at the surface through dipole–dipole and van der Waals interactions.	[46]
	Ovalbumin (OVA)	Binding occurs spontaneously and is exothermic, showing a reduced affinity at elevated temperatures (K = 1.41 × 10^4^ at 298 K to 2.39 × 10^3^ L/mol at 318 K). High quenching constants (Kq ≈ 10^13^ L.mol^−1^·s^−1^) suggest that static quenching is prevalent. The interaction is primarily stabilized by hydrogen bonding and van der Waals forces (ΔH = −108.9 kJ/mol; ΔS = −286 J/mol.K).	[47]
	Human serum albumin (HSA)	Binding takes place through a static quenching mechanism involving a single binding site (Kb = 4.47 × 10^6^ M^−1^; n ≈ 1). A significant quenching rate constant (Kq = 9.3 × 10^13^ M^−1^.s^−1^) validates the formation of a ground-state complex. Förster energy transfer analysis reveals a donor–acceptor distance of 3.19 nm (R_0_ = 2.73 nm; E = 0.28). The interaction is concentrated around Trp214, mainly within a hydrophobic cavity.	[48]
	β-Casein (BCN), β-Lactoglobulin (BLG), Bovine serum albumin (BSA)	Resveratrol exhibits a strong binding affinity to all three proteins, with Stern–Volmer constants recorded at 7.33 × 10^5^ M^−1^ for BCN, 4.64 × 10^5^ M^−1^ for BSA, and 8.25 × 10^5^ M^−1^ for BLG. The quenching rate constants, approximately ranging from 10^13^ to 10^14^ M^−1^.s^−1^, surpass the diffusion limit, which suggests the occurrence of static quenching. The accessibility of the fluorophore was found to be greatest for BLG at 82%, followed by BSA at 67%, and BCN at 60%.	[49]
	Peanut arachin (ARA, 11S globulin)	Binding takes place through a static quenching mechanism, with Kq values ranging from 6.53 to 8.17 × 10^12^ L.mol^−1^s^−1^, which surpass the diffusion limit, suggesting the formation of a ground-state complex. The binding constant is approximately 1 × 10^5^ M^−1^, indicating around 1 site (n ≈ 1). Thermodynamic data reveal that the interaction is spontaneous, endothermic, and driven by entropy (ΔH > 0; ΔS > 0; ΔG < 0), primarily influenced by hydrophobic forces, along with contributions from van der Waals interactions and hydrogen bonding.	[50]
	Phosvitin (egg yolk phosphoprotein)	The process of binding is characterized as spontaneous and endothermic, with a Gibbs free energy change (ΔG) ranging from −24 to −27 kJ/mol, an enthalpy change (ΔH) of +53.5 kJ/mol, and an entropy change (ΔS) of +261 J/mol.K. This phenomenon is primarily driven by hydrophobic interactions, although hydrogen bonding also plays a role. Furthermore, the binding affinity is observed to increase with temperature, with equilibrium constants (K) varying from 1.8 × 10^4^ to 3.6 × 10^4^ L/mol, indicating approximately one binding site (n ≈ 1.0).	[51]
	Rice glutelin (RG)	Quenching encompasses both static and dynamic mechanisms. The constants for dynamic quenching exhibited a decrease with rising temperature (KD = 1.15 × 10^5^ to 3.76 × 10^4^ M^−1^), while the corresponding kqD values remained above the diffusion limit. Static binding demonstrated a high affinity (Ks = 1.01–1.28 × 10^6^ M^−1^) with approximately one binding site (n ≈ 0.5–0.8). The binding process was spontaneous (ΔG ≈ −39 to −42 kJ/mol), exothermic (ΔH = −3.34 kJ/mol), and driven by entropy (ΔS = +124.8 J/mol.K). The primary residues involved in quenching were tyrosine residues.	[52]
	Bovine trypsin	Binding occurs through a static quenching mechanism characterized by a single binding site (n ≈ 1) and an increased affinity at elevated temperatures (K = 0.9–3.2 × 10^5^ M^−1^ from 298–310 K). The interaction is spontaneous (ΔG = −28.3 to −32.6 kJ/mol), driven by entropy (ΔH = +80.2 kJ/mol; ΔS = +364 J/mol.K), and is mainly influenced by hydrophobic interactions. A red shift in fluorescence suggests a conformational loosening around Trp/Tyr residues.	[53]
	Catalase	Binding occurs spontaneously and is exothermic (ΔG = −16.9 to −20.4 kJ/mol; ΔH = −31.96 kJ/mol; ΔS = −50.7 J/mol.K), with approximately one binding site (n ≈ 1.0). Affinity exhibits a slight increase with temperature (KA = 9.67 × 10^3^ to 2.12 × 10^4^ L/mol), whereas Stern–Volmer constants show a decrease. Förster analysis indicates a donor–acceptor distance of 4.92 nm. The stabilization of binding is primarily attributed to van der Waals forces and hydrogen bonding.	[54]
	Pancreatic lipase (LPS)	Binding occurs spontaneously and is exothermic (ΔG = −40.4 to −33.5 kJ/mol; ΔH = −44.3 kJ/mol; ΔS = −135.5 J/mol.K), mainly influenced by hydrogen bonding and van der Waals forces. The affinity exhibits a slight decrease with increasing temperature (KA = 7.5 × 10^5^ to 1.2 × 10^5^ L/mol), with approximately one binding site (n ≈ 1.2–1.3).	[55]
	Human insulin (chain B, bovine pancreas)	The interaction involves a combination of static and dynamic quenching mechanisms. KD ranged from 3.03 × 10^4^ to 11.5 × 10^4^ M^−1^, with kqD values on the order of 10^13^ M^−1^·s^−1^. The dynamic component was endothermic and non-spontaneous (ΔH = +19.7 kJ/mol; ΔS = +23.8 J·mol^−1^·K^−1^; ΔG > 0). Static quenching yielded Ks on the order of 10^5^ M^−1^ with kqS values exceeding 10^14^ M^−1^·s^−1^, confirming ground-state complex formation. The number of binding sites was approximately one (n ≈ 0.5–0.8). The static binding component was spontaneous (ΔG ≈ −38.7 to −41.8 kJ/mol), slightly exothermic (ΔH = −3.34 kJ/mol), and primarily driven by entropy (ΔS = +124.8 J·mol^−1^·K^−1^), indicating hydrophobic interactions as the dominant stabilizing force. The tyrosine residues of insulin chain B were identified as the primary fluorophores involved in the quenching process.	[56]
	DAPK1 kinase domain (residues 1–285)	The binding is direct and depends on concentration, exhibiting an apparent dissociation constant (Kd, app) of 8.5 ± 1.9 µM, which was determined through tryptophan fluorescence quenching at 340 nm using the Langmuir model	[42]
	Calf thymus DNA (ctDNA) using acridine orange (AO) as probe	Resveratrol inhibited AO–ctDNA fluorescence through a static quenching mechanism. The Stern–Volmer quenching constants are Ksv: 2.38 × 10^4^ L/mol (299 K), 2.24 × 10^4^ (310 K), and 2.01 × 10^4^ (315 K). The binding constant is K = 5.49 × 10^3^ L/mol (17 °C) and 1.90 × 10^4^ L/mol (37 °C). The thermodynamic parameters at 37 °C are: ΔH = 46.4 kJ/mol, ΔS = 231.8 J K^−1^·mol^−1^, and ΔG = −25.4 kJ/mol. These results indicate an intercalation binding mode between resveratrol and ctDNA.	[34]
**Circular dichroism**	β-Lactoglobulin (β-LG)	The binding of resveratrol did not influence the secondary structure of β-lactoglobulin; however, it did induce alterations in the environment surrounding aromatic residues, suggesting a disturbance in the tertiary structure.	[57]
	β-lactoglobulin (BLG), bovine serum albumin (BSA), β-casein (BCN)	The binding of resveratrol did not affect the secondary structures of proteins: β-lactoglobulin preserved its β-sheet characteristic (~212 nm), BSA sustained its α-helical bands (209 and 221 nm), and the BCN spectrum showed no alterations.	[49]
	Insulin (bovine pancreas)	Far-UV circular dichroism (CD) demonstrated a reduction in α-helix content from 77.1% to 68.1% following the binding of resveratrol. Near-UV CD exhibited a diminished intensity at 275 nm, suggesting the disruption of disulfide bonds and the depolymerization of insulin dimers.	[58]
	Human serum albumin (HSA) and hemoglobin (Hb)	Resveratrol decreased the α-helix content in human serum albumin (HSA) from 49.7% to 40.3% (−18.75%) and in hemoglobin from 36.6% to 33.1% (−9.43%). Nevertheless, the circular dichroism (CD) spectra preserved the typical α-helical configuration, suggesting a degree of unfolding while avoiding total structural collapse.	[59]
	Bovine serum albumin (BSA) in the presence of β-cyclodextrin (β-CD)	BSA preserved its mainly α-helical secondary structure when binding with resveratrol, even in the presence of β-cyclodextrin	[39]
	Lipase (LPS)	Resveratrol caused minimal alterations in the secondary structure of LPS, resulting in slight reductions in α-helix (from 17.2% to 17.0%), β-sheet (from 43.1% to 42.6%), and β-turn (from 20.9% to 20.8%), alongside a minor increase in random coil content (from 41.6% to 42.9%).	[55]
	Insulin (bovine, chain B oxidized)	CD spectra indicated shifts in peaks from 209 to 211 nm and from 217 to 219 nm, accompanied by a broadened band at 222 nm. Analysis of the secondary structure demonstrated a minor increase in the length of α-helices and a slight reduction in β-sheet content.	[56]
	Bovine serum albumin (BSA)	The binding of resveratrol resulted in a slight decrease in the α-helical structure of BSA, without significant disruption to the overall secondary structure (the α-helix content diminished marginally from 61.3% (BSA) to 59.8% (BSA–RES)).	[60]
	β-Lactoglobulin (β-LG)	The binding of resveratrol led to a partial destabilization of β-LG by decreasing the percentage of β-sheets from 49% to 44% and increasing the percentage of turns from 15% to 19%.	[61]
	Human serum albumin (HSA)	Resveratrol enhanced the stability of human serum albumin (HSA) by elevating the α-helical content to 65.1% at a concentration of 10 µM and to 68.2% at 20 µM, while also raising the thermal melting point from 63.8 °C for HSA to 66.3 °C when combined with resveratrol.	[62]
	Bovine liver catalase (BLC)	Resveratrol enhanced the α-helix content by approximately 1% while reducing the β-sheet content by about 1%, with minimal alterations observed in the β-turn and random coil structures.	[63]
	β-Lactoglobulin (β-LG, native and ultrasound-pretreated 0–5 min)	Resveratrol induced minimal structural alterations: the α-helix fluctuated between 15.5% and 16.7%, the β-strand ranged from 30.7% to 31.8%, β-turns were observed at 21.5% to 22.1%, and unordered structures varied from 30.6% to 31.1%, irrespective of ultrasound pretreatment. The β-sheet characteristic at approximately 215 nm remained intact.	[64]
	α-Lactalbumin (α-LA)	Resveratrol reduced the α-helix content from 33.3% to 29.1% and the β-sheet from 30.2% to 29.6%, whereas the unordered coil increased from 30.4% to 35.1%. In the ternary system comprising α-LA, curcumin, and RES, the α-helix further decreased to 25.8%, while the unordered coil increased to 41.4%	[65]
	Insulin (bovine, chain B oxidized)	The results from the CD spectropolarimeter indicated that incorporating resveratrol influenced the secondary structure of insulin. There was a slight alteration and increase in the amount of α-helix and β-sheet, as well as in the length of the secondary structure.	[56]
**DSC**	Human serum albumin	Differential scanning calorimetry indicated elevated melting temperatures (Tm1 increased from 56.9 to 58.2 °C; Tm2 increased from 64.6 to 67.1 °C at a 1:1 ratio) and a rise in unfolding enthalpy (ΔH increased from 634 to 729 kJ·mol^−1^). The stabilization effect was particularly significant in the ligand-binding domain.	[66]
	α-Lactalbumin	DSC identified two peaks of denaturation. In the presence of resveratrol, T1 (the non-α-helix domain) exhibited an upward shift (from 333 K to 336 K), suggesting a stabilization effect, whereas T2 (the α-helix domain) demonstrated a downward shift (from 355 K to 351 K), indicating destabilization. The calorimetric enthalpy change (ΔHcal) increased from 100 kJ/mol to 106 kJ/mol, and the entropy change (ΔS) showed a slight increase. In summary, resveratrol has a dual impact: it stabilizes the initial stages of unfolding while facilitating the denaturation of the α-helix in later stages.	[67]
	Phosphatidylcholine liposomes	DSC analysis revealed that both resveratrol and piceid caused a concentration-dependent broadening and reduction in phase transition peaks. The observed peak broadening and shifts in Tm indicate a decrease in cooperativity and interfacial localization of the antioxidant resveratrol	[68]
	Commercial lipid mixture P90NG	DSC analysis revealed that resveratrol induced a concentration-dependent reduction in the primary transition temperature of DPPC liposomes (Tm decreased from 41.3 °C to 38.3 °C at a 0.5 molar ratio) and inhibited the pretransition peak. The primary transition peak exhibited broadening, and its enthalpy diminished (ΔH reduced from 34.5 to 28.7 kJ/mol), which indicates a decrease in bilayer cooperativity. These observations imply that resveratrol intercalates within the vicinity of the lipid head-group region, thereby disrupting membrane organization and enhancing fluidity.	[69]
	DOPC and DOPC/Chol (4:1) membranes (MLVs)	In pure DOPC bilayers, resveratrol caused a decrease in the main transition temperature (Tm from −16.8 °C to −24.5 °C at a lipid: RSV ratio of 4:1) and a reduction in enthalpy (ΔH from 10.3 to 2.8 kcal/mol), with saturation observed at elevated RSV concentrations. In DOPC/Chol (4:1), the shifts in Tm were less pronounced (−18.9 °C to −27.9 °C), yet the reduction in enthalpy was more significant (ΔH from 4.1 to 0.9 kcal/mol). At lipid: RSV ratios of 30:1 or greater, the splitting of thermogram peaks suggested the occurrence of phase separation.	[70]
	Phosphatidylcholine (DMPC and DPPC) model membrane	Resveratrol caused a concentration-dependent reduction in the primary transition temperature of DMPC (ΔTm reaching −4 °C) and DPPC (ΔTm reaching −2.7 °C) at a 0.12 molar ratio. The impact on transition enthalpy was minimal and inconsistent. As the resveratrol concentration increased, the transition peaks became broader; in DMPC, elevated ratios led to peak asymmetry, indicating overlapping transitions.	[71]
	DMPC membranes (multilamellar vesicles)	Resveratrol altered the thermotropic characteristics of DMPC, removing the pretransition phase and reducing as well as broadening the main transition temperature. At resveratrol molar fractions of 0.09 or greater, DSC demonstrated peak splitting, which suggests phase separation into domains that are rich in resveratrol and those that are poor in resveratrol.	[72]

**Table 2:** Summary of in silico studies examining resveratrol–carrier and resveratrol–target interactions. The table compiles representative computational investigations, including molecular docking and molecular dynamics (MD) simulations, focused on the structural, energetic, and dynamic aspects of resveratrol binding. Reported parameters include binding affinities, key interacting residues, hydrogen-bond and hydrophobic interaction profiles, and predicted binding energies (ΔG). The collected data provide a comparative overview of computational approaches used to elucidate resveratrol’s conformational behavior, binding mechanisms, and stabilization within biological and carrier systems.

Computational Method	Target/Carrier	Key Finding	Ref
Molecular docking	HSA	Docking located trans-resveratrol at HSA Sudlow’s site I (subdomain IIA) with −7.76 kcal/mol, stabilized mainly by hydrogen bonding and van der Waals interactions, with Trp-214 identified as critical for complex stability.	[62]
Molecular docking	Whey protein isolate (β-LG and α-LA)	Docking placed resveratrol in hydrophobic cavities of beta lactoglobulin and alpha lactalbumin, stabilized by hydrophobic contacts and hydrogen bonds, with stacking involving Tyr20 and Tyr103.	[32]
Molecular docking	α- and β-Caseins	The docking process positioned resveratrol in α casein close to Leu142, Tyr144, Phe150, and Pro147, as well as in β casein near Asn7, Leu3, Phe87, Leu88, Pro115, and Val116, yielding free binding energies of −11.56 and −12.36 kcal/mol, respectively.	[101]
Molecular docking + Molecular dynamic simulation	β-Lactoglobulin	Docking located resveratrol in β-LG hydrophobic cavity with H-bonds to Ser21, Gln35, Val43, Cys160; MD with MM/PBSA revealed a stable complex with ΔG of −27.6 kcal/mol and RMSD between 0.22 and 0.25 nm	[102]
Molecular docking + Molecular dynamic simulation	β-Lactoglobulin (β-LG) with resveratrol ± curcumin	Docking positioned resveratrol on the upper surface of beta lactoglobulin through hydrophobic interactions; in the presence of curcumin, competition diminished the affinity of resveratrol, and 100 ns MD indicated that resveratrol remained close to the entrance of the barrel while the complexes maintained stability.	[103]
Molecular docking + Molecular dynamic simulation	β-Cyclodextrin inclusion complex	Docking placed resveratrol inside β-cyclodextrin with a −23.25 kcal/mol score and three H-bonds from the resorcinol ring to glucose OH groups, and 100 ns MD showed a stable, more compact inclusion complex (stable RMSD, slight β-CD flexibility, decreased Rg and SASA) maintaining up to three H-bonds	[104]
Molecular docking	β-Cyclodextrin–resveratrol complex at lipid bilayer	MD simulations of CD–resveratrol complexes (1:1) identified four motifs—M-form (head-first, most stable; −99.574 kJ/mol), D-form (tail-first; −96.823 kJ/mol), head-to-multi insertion in γ-CD, and edge-form surface adsorption (weakest; −45.527 kJ/mol), with ESP distribution analyses explaining the head-first preference.	[105]
Molecular docking	Insulin	Docking placed resveratrol between insulin helices A and B, stabilized by H-bonds with Asn21 and Gly24, π–π stacking with Tyr19 and Tyr26, and van der Waals contacts with Asn18, Phe25, Cys20, giving affinity −6.3 to −5.4 kcal/mol across nine conformations.	[56]
Molecular docking	Giardia lamblia tubulin (α/β heterodimer)	Docking placed resveratrol in the hydrophobic α/β-tubulin interface pocket (podophyllotoxin site), contacting β-tubulin Lys350 and residues Leu246, Lys252, Asn256, Val351, Ser352, and α-tubulin Ala178, Thr179, supporting a high-probability binding near the heterodimer interface.	[106]
Molecular docking + Molecular dynamic simulation	Promoter DNA d(CCAATTGG)2 (oncogene motif)	Docking positioned resveratrol in the AT minor groove of d(CCAATTGG)2; 2 ns MD gave a stable complex with RMSD about 0.55 Å.	[88]
Molecular docking	Short DNA sequences d(CGTTAACG)2 and d(CGAATTCG)2	Docking placed resveratrol in the minor groove at central AT rich AATT or TTAA segments of two DNA models, with binding energies −8.35 and −9.53 kcal/mol, stabilized by hydrogen bonds.	[107]
Molecular dynamic simulation	Phospholipid membrane	MD demonstrated that resveratrol enhances the area per lipid while reducing membrane thickness; it favored a shallow position near headgroups that form hydrogen bonds with ester groups.	[30]
Molecular docking + Molecular dynamic simulation	MAPK/EGFR (and other core targets)	Docking/MD revealed significant interactions between resveratrol and key targets, including MAPK and EGFR.	[108]
Molecular docking + Molecular dynamic simulation	Monoamine oxidase A (MAO-A)	In silico analysis suggested resveratrol can occupy a specific allosteric pocket between helices H3 and H24 in MAO A; 300 ns MD supported binding stability.	[109]

## Data Availability

The original contributions presented in this study are included in the article. Further inquiries can be directed to the corresponding author.
